# Resistance Training and Older Adults with Type 2 Diabetes Mellitus: Strength of the Evidence

**DOI:** 10.1155/2012/284635

**Published:** 2012-09-04

**Authors:** Nina Hovanec, Anuradha Sawant, Tom J. Overend, Robert J. Petrella, Anthony A. Vandervoort

**Affiliations:** ^1^Health and Rehabilitation Sciences Graduate Program, Western University, London, ON, Canada N6G 1H1; ^2^School of Physical Therapy and Center for Physical Activity and Aging, Faculty of Health Sciences, Western University, London, ON, Canada N6G 1H1; ^3^Department of Family Medicine, Western University, London, ON, Canada N6G 1H1

## Abstract

*Objective*. This paper analyzes the effects of resistance training (RT) on metabolic, neuromuscular, and cardiovascular functions in older adults (mean age ≥ 65 years) with type 2 diabetes (T2DM). *Research Design and Methods*. A systematic review conducted by two reviewers of the published literature produced 3 records based on 2 randomized controlled trials that assessed the effect of RT on disease process measures and musculoskeletal/body composition measures. Statistical, Comprehensive Meta-Analysis (version 2) software was used to compute Hedge's g, and results were calculated using the random effects model to account for methodological differences amongst studies. *Results*. Largest effect of RT was seen on muscle strength; especially lower body strength, while the point estimate effect on body composition was small and not statistically significant. The cumulative point estimate for the T2DM disease process measures was moderate and statistically significant. *Conclusions*. RT generally had a positive effect on musculoskeletal, body composition, and T2DM disease processes measures, with tentative conclusions based on a low number of completed RCTs. Thus, more research is needed on such programs for older adults (≥65 years) with T2DM.

## 1. Introduction 

Type 2 diabetes mellitus (T2DM) in older adults is an emerging epidemic [1]. (For the purpose of this paper, the term “older adults” refers to individuals who are at least 65 years old.) It is an age-prevalent metabolic disorder, characterized by insulin resistance with relative insulin deficiency [2, 3], with the highest prevalence found in individuals who are 80 years or older—an estimated number of 40 million is expected in the United States by the year 2050 [1].

Physical activity is considered to be a cornerstone of T2DM prevention and management [2, 4], and it is important to have accurate information for health care organizations to integrate into their knowledge management strategies [5]. Physical activity refers to “the expenditure of energy above that of resting by contraction of skeletal muscle to produce bodily movement,” while exercise is “a type of physical activity that involves planned, structured and repetitive bodily movement performed for the purpose of improving physical fitness” [6, page 359]. Physical activity and exercise will be used interchangeably in this paper. 

In terms of physical activity as a management method in populations living with T2DM, traditional focus has been given to aerobic training (AT) interventions [7, 8]. Aerobic training activates large muscle groups to perform activities such as swimming and running, increasing the function of the heart, lungs, and muscle mitochondria to meet the heightened oxygen demands, ultimately resulting in cardiorespiratory fitness improvements [9]. Over the past decade, interest has also emerged in conducting studies that assess the potential effect of resistance training (RT) interventions in older individuals with T2DM [10–12]. Resistance training activates the muscular system to generate force against a resistive load [4]; it can be performed by utilizing various exercise machines, lifting free-weights (e.g., dumbbells), or doing calisthenics such as situps, pushups, crunches, and lunges. If RT is performed regularly, where the weight lifted is increased to moderate (50% of 1RM (1RM represents 1 Repetition Maximum, which refers to the maximum weight that a person can lift once)) and high levels of intensity (>75% 1RM), it often leads to increased muscle mass and improvements in muscular fitness [4, 13–15]. Muscular fitness refers both to muscle strength, the amount of force produced by a muscle, and muscle endurance, the ability of a muscle to “exert submaximal force for an extended period of time” [16, page 27]. 

Resistance training may be more appealing and feasible than AT for people with T2DM who are often overweight and sedentary [17], as well as for older adults, obese, and/or frail individuals [4, 12, 18]. With advanced age, there is a significant loss of muscle mass and strength, a phenomenon known as sarcopenia [19]. It has recently been indicated that older adults with T2DM tend to have greater muscle mass loss, worse muscle quality (defined as the amount of muscle strength per unit of regional muscle mass), reduced upper and lower body strength, greater visceral adipose content, as well as higher risk for functional decline and disability than their healthy, age-matched counterparts [20–24]. Resistance training might benefit older adults living with T2DM through muscle hypertrophy, enhanced muscle quality, strength gains for greater power development with more effective mobility function, and glycemic profile improvements [25].

**Figure 1:**
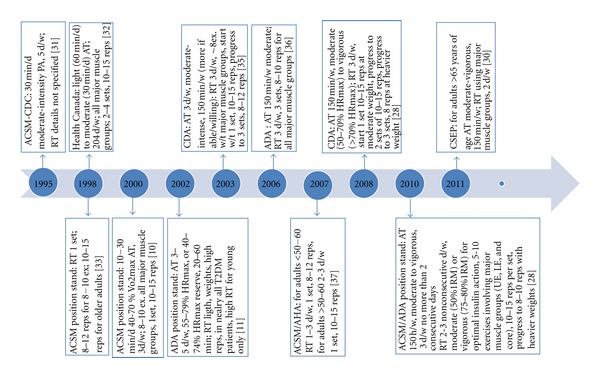
Chronological Timeline of PA Recommendations for T2DM from Various Professional Organizations [modified from [26]]. PHAC [Public Health Agency of Canada]; CSEP [Canadian Society for Exercise Physiology]; CDA [Canadian Diabetes Association]; ACSM [American College of Sports Medicine]; ADA [American Diabetes Association]; CDC [Centers for Disease Control and Prevention]; AHA [American Heart Association]. PA [Physical Activity]; RT [resistance training]; AT [aerobic training]; UE [upper extremity]; LE [lower extremity]; HR_max⁡_ [maximum heart rate]; VO_2max⁡_ [maximal oxygen uptake/consumption]; d [days]; w [week]; w/t [with]; reps [repetitions]; ex [exercises]; h [hour]; min. [minute].

Resistance training studies in populations with T2DM were not readily available prior to 1997 [4]. The first physical activity guidelines specifically designed for adults with T2DM were developed by the American College of Sports Medicine (ACSM) in the year 2000 [10]. As illustrated in Figure 1, a modified timeline first introduced by Hills and colleagues in 2010 [26], agencies such as the Canadian Diabetes Association (CDA), the American Diabetes Association (ADA), the Canadian Society for Exercise Physiology (CSEP), and ACSM now include RT recommendations within their physical activity guidelines [11, 27–37].

Due to the associated increases in blood pressure (BP) that may be harmful, there could be unsubstantiated apprehension in recommending RT, especially at higher intensities. The main concern is that these BP increases could lead to a stroke, myocardial ischemia, or retinal hemorrhage [4]. This may partially explain the historical dominance of AT interventions in populations living with T2DM. However, there is a lack of scientific evidence that RT actually increases any of the aforementioned risks, as no RT-related adverse events have been reported in studies where individuals with T2DM were assessed [4, 38]. Additionally, past researchers have suggested that RT may actually reduce BP levels [39–41]. Finally, there are precautions that can be employed to avoid potentially harmful side-effects of exercise, such as avoiding physical activity under certain circumstances (detailed by Gordon in 2002 [7]) and conducting appropriate preexercise screens and assessments [7, 35, 42].

Skeletal muscles are the largest postprandial glucose uptake and glycogen storage sites in the human body and as such are integral in maintaining glucose homeostasis. Resistance training may reverse or at least limit some of the aforementioned negative neuromuscular effects associated with aging and/or T2DM [43]. Previous meta-analyses have reported benefits of aerobic training, resistance training, or a combination of the two on reducing HbA1c levels, which signifies improved glycemic control [25, 38, 44–47]. A recent meta-analysis demonstrated that supervised aerobic or resistance training led to greater declines in HbA1c levels than exercise advice only [44]. However, no previous meta-analysis has assessed the effects of RT in older adults (≥65 years) with T2DM. At this time, the literature base may benefit from such a review, since older adults often experience detrimental neuromuscular and sensorimotor changes associated with aging (e.g., sarcopenia) placing them at an increased risk for mobility problems, injury from falls, and disability [21, 48]. Furthermore, T2DM is most common in older adults, who as a result of this disease often experience various comorbidities [49], further reducing their capacity to live independently (e.g., retinopathy, which may lead to blindness; peripheral neuropathy, which may lead to foot ulcers and amputations; nephropathy, which over time could result in renal failure, etc.). Thus, the purpose of this paper is to conduct a systematic review of the best available evidence, in order to assess the effect of RT on metabolic, neuromuscular, and cardiovascular functions in older adults with T2DM.

## 2. Methods

 This meta-analysis utilized the PRISMA as a framework when selecting studies for inclusion in this paper [50]. This meta-analysis is not registered with any institution, such as the Cochrane Collaboration. The literature search was conducted until the end of August 2011, using electronic databases (Medline, EMBASE, AMED, PubMed, Scopus, CINAHL) that generated MESH terms based on the following keywords: resistance training, type 2 diabetes, and aged. The search terms were entered into the databases using the appropriate combinations of “OR” and “AND.” In order for articles to be included in this paper, the following inclusion and exclusion criteria needed to be satisfied.

*Inclusion Criteria*

- RCTs.
- Published between the years 2000 and 2011.
- RT interventions or a combination of RT and other forms of intervention (e.g., flexibility, weight loss, standard care, etc.).
- Participants with established T2DM.
- Participants' mean age ≥65 years.

*Exclusion Criteria*

- Participants with the presence of another chronic illness (e.g., cancer).
- Non-English publications.
- Studies reporting effect of RT in previously trained participants.
- Studies reporting effect of RT on outcome measures not relevant to this paper (see Table 1 for all relevant outcome measures).

The aforementioned inclusion and exclusion criteria were developed in order to obtain the most recent (2000–2011), scientifically rigorous (RCTs) evidence on the specific effect of resistance training in older adults with type 2 diabetes. Various studies, review articles, and commentaries that did not satisfy the inclusion criteria were used to inform the introduction and the discussion sections of this paper. Furthermore, NH and AS independently reviewed and rated the articles and any differences were resolved by discussion or by comparison to the ratings provided on the PEDro website. To limit redundancy, Cohen's Kappa values were not calculated since there were no major disagreements between the authors (i.e., >95% agreement).

**Outcome Measures**

The primary outcome measures were grouped into three major areas including body composition, musculoskeletal, and type 2 diabetes disease process measures. Table 1 summarizes the major outcome headings and their respective measures.

**Methodological Quality of the Studies**

Internal validity of studies included in this paper was assessed using the PEDro scale—a valid [51] and reliable [52] tool to evaluate study quality. Article ratings are included as PEDro scores listed in Table 3, while rating criteria are detailed in Table 5.

**Statistical Analyses**

Statistical software (Comprehensive Meta-Analysis—version 2) for meta-analysis of binary, continuous, and diagnostic data was used for computation of Hedge's *g* (a measure of effect size). Hedge's *g* values were used to assess the influence of strengthening exercises on body composition, musculoskeletal measures, and type 2 diabetes disease outcomes (previously summarized in Table 1). The effect sizes were interpreted as small, medium and large if they were 0.2, 0.5, and 0.8, respectively [54]. A 95% confidence interval was constructed around the point estimate of the effect size. Any standard errors that were reported by study authors were converted to standard deviations using the formula SD = *√n*∗SE, where SD is the standard deviation, *√* is the square root symbol, *n* refers to the sample size, ∗ represents the multiplication function, and SE is the standard error [55].

The statistical significance of the differences in the effects of RT on body composition, muscle quality, and strength along with moderator variables included for the effect on disease processes was computed by Page's *L* statistic with the use of PASW 18 statistical software to calculate the sum of squares (SS) between groups, as well as total SS. Page's *L* statistic was then calculated using the formula *L* = [*N* − 1]*r* ^2^, where *N* is the total number of effect sizes and *r* ^2^ is the product of SS_between_/SS_total_. (Further details regarding Page's *L* statistic can be found in [56]) When performing meta-analysis, the overall effect of an intervention can be influenced by use of particular outcome measures or intervention strategies. Page's *L* statistics was utilized to elucidate such differences in the current study.

The presence of heterogeneity among the moderator variables was evaluated by the *Q* statistic using a random effects model. The studies were considered heterogeneous if the *P* value of the *Q* statistic was <0.1, which has been proposed as the appropriate alternative to the conventional *P* < 0.05, when there is a low number of articles included in a review [57]. Publication bias was not assessed, since there were only three articles included, and any conclusions that are drawn from the results that emerge from this meta-analysis cannot be taken as definitive. The robustness of the findings was established based on the assessment of the effect size and its associated confidence intervals, rather than other methods, such as the calculation of Fail Safe N, which can lead to widely varied estimates [58]. The results reported were calculated using the random effects model, in order to account for methodological differences amongst studies. The statistical significance for the effect sizes' statistical tests (i.e., Hedge's *g*) was set at *P* < 0.05. 

## 3. Results

**Figure 2:**
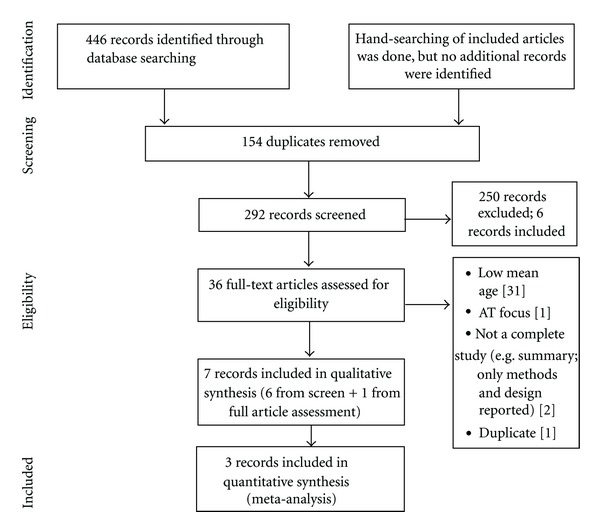
Study selection diagram [50] AT-aerobic training.

Three [13, 17, 53] of the 446 citations were included in the final analysis (Figure 2). However, 2 of the citations [13, 17] are technically considered one study, since their findings are based on the same pool of participants, but they are both included in the meta-analysis since each of them provides relevant but different outcome measures. A total of 32 effect sizes, evaluating the effect of strength training on the disease process (20 effect sizes) and muscle quality (12 effect sizes), were extracted from the included studies. Participant and study characteristics are described in Tables 2 and 3 respectively.

### 3.1. Effect of RT on T2DM Disease Process Measures

Serum insulin [17, 53], HbA1c [17, 53], HDL [13, 53], LDL and total cholesterol [13, 53], fasting glucose [17, 53], and BP [13, 53] were analysed to evaluate the effect of RT on the disease process. The overall cumulative point estimate of this effect size was statistically significant (Hedge's *g* = −0.246; *P* = 0.023; 95% CI: −0.458, −0.034). 

 For individual variables, the effect of RT on BP (Hedge's *g* = −0.540; *P* < 0.001; CI: −0.832, −0.248), insulin (Hedge's *g* = 0.505; *P* = 0.016; CI: 0.094, 0.916), total cholesterol, and LDL cholesterol (Hedge's *g* = 22120.464, *P* = 0.002; CI: −0.760, −0.169) was statistically significant. However, the effect of RT on fasting glucose (Hedge's *g* = −0.121; *P* = 0.559; CI: −0.526, 0.284), HbA1c (Hedge's *g* = −0.463; *P* = 0.145; CI: −1.084, 0.159), and HDL cholesterol (Hedge's *g* = 0.134; *P* = 0.517; CI: −0.271, 0.539) was not as consistent between studies in terms of magnitude of improvement and fluctuations in control group. Also, the differences in effects of RT on fasting glucose, insulin, HBA1c, cholesterol, HDL, FFA, and BP were not statistically significant (L(19) = 14.109; *P* > 0.05).

### 3.2. Effect of RT on Body Composition Measures

Lean body mass [17, 53] and fat body mass [53, 59] were analysed to evaluate the effect of RT on body composition. The cumulative point estimate effect of RT on body composition was small but not statistically significant (Hedge's *g* = 0.199; *P* = 0.197; CI: −0.103, 0.500). The effect of RT on lean body mass (Hedge's *g* = 0.395; *P* = 0.220; CI: −0.237, 1.028) was larger than on fat body mass (Hedge's *g* = 0.066; *P* = 0.749; CI: −0.339, 0.471), but neither was statistically significant. 

### 3.3. Effect of RT on Musculoskeletal Measures

Whole body, lower and upper body muscles strength [13, 53], and muscle quality were analysed to evaluate the effect of RT on overall muscle strength and quality. The cumulative point estimate effect of RT on muscle strength (Hedge's *g* = 1.05; *P* < 0.001; 95% CI: 0.699, 1.404) and overall quality (Hedge's g = 0.816 *P* = 0.008; 95% CI: 0.216, 1.415) were large and statistically significant. The largest effect of RT was on lower body strength (Hedge's *g* = 1.415; *P* < 0.001; CI: 0.864, 1.967), followed by upper body strength (Hedge's *g* = 0.974; *P* < 0.001; CI: 0.453, 1.494), and both were statistically significant. The effect of RT on whole body strength was also large and statistically significant (Hedge's *g* = 0.802; *P* = 0.002; CI: 0.291, 1.313). 

The effect of RT on muscle quality (Hedge's *g* = 1.460; *P* < 0.001; CI: 0.906, 2.015) was large and statistically significant. The differences in effect of RT on body composition, muscle quality, and strength were not statistically significant (L(11) = 13.762; *P* > 0.05). However, the CI ranges were wide for all measures (musculoskeletal, disease process, and body composition); as such any conclusion drawn based on the effect sizes and statistical significance needs to be considered with caution. 

The heterogeneity (*Q*-values with their respective df and *P* values) for all moderator variables is summarized in Table 4. However, the number of studies included in the analysis is too small to infer definitive conclusions regarding heterogeneity.

## 4. Discussion

The purpose of this paper was to conduct a systematic review and meta-analysis of the currently available evidence, in order to assess the effect of resistance training in older adults with T2DM. The findings generally show that RT has an effect on the musculoskeletal system, disease process, and body composition to varying degrees (see Table 6 for a summary of the outcome measures, their respective effect sizes, and statistical significances). Overall, RT had the largest effect on the musculoskeletal measures, followed by disease process measures, while the smallest effect was seen on the body composition measures. 

It is not surprising that RT had the largest effect on musculoskeletal measures, as it is a well-established mode of exercise to induce neuromuscular changes, such as increased muscle size and strength [15]. Specifically, findings from this analysis indicate that RT increases muscle strength and quality. These effects could be quite consequential for the investigated population, as aging and T2DM are linked with reduced muscle mass and strength, increased adiposity, and a sedentary lifestyle [12]. 

Although the underlying molecular causes of T2DM are unknown, it has been associated with obesity, visceral adiposity, and physical inactivity, which all contribute to an increased risk of developing cardiovascular disease and various disabilities [2, 23, 24]. As such, older adults with T2DM are placed at “double jeopardy” with regards to their health status, which greatly increases their dependence on health care services [1]. A large US-based, cross-sectional study illustrated this point when older adults (70–79 years) with and without T2DM were compared [1]. Various publications from this study showed that those with T2DM had lower muscle strength and quality [21], accelerated muscle loss (i.e., loss of knee extensor strength at a more rapid rate), and excessive muscle mass loss (i.e., greater loss in the amount of leg lean mass) when compared with healthy, age-matched counterparts [20, 22]. Reductions in muscle strength and quality have been linked to an increased risk of physical disability, such as mobility problems and falls [48]. Findings from the current meta-analysis suggest that muscle strength and quality improvements in older adults with T2DM could induce greater functional capacity and reduce the risk of disabilities. Furthermore, muscle quality and strength gains may result in greater physical activity participation in various populations [60–62], including older adults with T2DM [13], which could in turn improve this populations' overall health status by reducing negative disease outcomes. 

In addition to improvements in muscle quality (the measure of strength per unit of muscle mass), one study that was included in this meta-analysis reported outcomes specifically regarding the cross-sectional area (CSA) of muscle fibers [17]. Although these outcome values could not be meta-analyzed since only one study included these measures, the fact that fiber hypertrophy resulted warrants further discussion. Brooks and colleagues showed that following a 16-week RT intervention the training group increased the CSA of type I and type II fibers, while the control group participants showed the opposite trend—a reduction in the CSA of both fiber types [17]. As well as strength gains leading to more effective force production, the increase in the CSA of muscle fibers, especially type I muscle fibers, might lead to a better delivery of oxygen through the greater capillary density and number of oxidative mitochondria [16]. In addition, these changes may improve the delivery of glucose from the blood to the muscle, while fiber hypertrophy may provide greater glycogen storage capacity within the muscles of individuals affected by T2DM and thus potentially improve insulin resistance [16, 17]. The hypothesis that muscle hypertrophy or larger muscle mass is associated with improved insulin sensitivity and glucose tolerance has previously been recognized [63]. 

A further elaboration may help to explain how RT might influence the interaction between the neuromuscular system and the underlying disease process of T2DM. Skeletal muscles represent the largest glucose deposition sites in the human body, which is negatively affected by insulin resistance—a defining feature of T2DM [64]. It has been suggested that people with T2DM have a defective insulin-dependent pathway, which is responsible for activating glucose transporters of the muscles to help move the glucose from the blood into the cells [65]. However, individuals with T2DM do not appear to have a flawed contraction-stimulated pathway for glucose transport [65]. For example, RT would induce a muscular contraction, in turn stimulating the translocation of the GLUT-4 (glucose transporter) to the tissue's cell membrane to dock and activate in order to accept the glucose molecules from the blood into the cell. Thus, glucose could enter the cell via this contraction-stimulated pathway even in individuals with T2DM whose insulin-dependent pathway is defective [65]. Furthermore, exercise has shown to increase GLUT4 expression in human skeletal muscle approximately two to four times, leading to improvements in glucose intolerance and insulin action [65–67]. This underlying mechanism may partially explain some of the effects of RT on the disease process outcomes in this meta-analysis. 

Resistance training also had some effects on various markers of the disease process associated with T2DM, including HbA1c, BP, fasting insulin, fasting glucose, HDL, total and LDL cholesterol. For example, findings from this meta-analysis indicated a nonsignificant, medium-sized effect of RT on reducing HbA1c, with a wide CI range. This could be the result of low sample size and a few studies; all of the results of this meta-analysis should be considered with caution. Nevertheless, reduction of HbA1c is considered one of the most important markers for glucose control, and a small change or improvement in this marker may result in a significantly reduced risk of developing diabetic comorbidities. Findings from a prospective study might help illustrate this point further, as decreasing HbA1c by 1% could reduce the risk of any diabetes-related complication by 21% [68]. Although this paper cannot confidently conclude that RT can effectively reduce HbA1c levels in older adults with T2DM, a previous meta-analysis by Boulé and colleagues was able to illustrate that RT was equally effective as AT at improving glycemic control in middle-aged adults [25]. On the other hand, recently Jorge and colleagues compared RT, AT, combined AT and RT, and a control group that received standard care [39]. They did not find significant reductions in HbA1c within any of the exercise groups when compared with the control group [39]. However, all groups had small sample sizes and the control group might have improved their diet during the time of the intervention while their standard care medication also could have contributed to the small difference between groups. Previous researchers have demonstrated that, in addition to RT effectively reducing HbA1c levels, it can also increase glucose disposal and storage capacity, improve lipid, as well as cardiovascular disease risk profiles in adults with T2DM [69–71]. 

This meta-analysis also showed a moderate effect of RT on BP, and a small effect on total and LDL cholesterol. However, the effect of RT on body composition measures, including lean body mass and fat mass, was small and nonsignificant. The positive effect of RT on BP and cholesterol may be promising, since achieving lower BP with exercise is indicative of improved cardiovascular function, while a reduction in cholesterol levels, especially LDL, may help reduce the risk of micro- and macrovascular complications, such as atherosclerosis, stroke, and myocardial infarction [2]. Past researchers have also found positive changes of BP that might have been induced by RT [39, 40]. These findings may be of considerable value for those with T2DM who have a two- to fourfold greater risk of developing cardiovascular disease [72]; improvements in LDL cholesterol as well as BP could improve health outcomes for this group. Improved physical function could lead to a greater ability to participate in various physical activities safely and enjoyably and in turn reduce the sedentary behavior often found in individuals with T2DM. However, some researchers did not find that RT led to a reduction in BP [53], nor improvements in the LDL cholesterol levels following AT, RT, or combined training [25]. Further studies are needed in order to better understand the potential effect of RT on BP and cholesterol in people with T2DM [53].

The fact that body composition was not altered may be due to the short intervention durations, or it could be attributed to the low number of studies included in this meta-analysis. However, despite RT apparently not having an effect on the body composition of older adults with T2DM, their metabolic control could still be impacted by exercise alone, since Boulé et al. indicated that RT and/or AT can enhance insulin sensitivity and glycemic control even when the weight and/or body composition is unaltered [25]. Future studies are needed to confirm this claim for older adults with T2DM. 

Although previous reviews indicate that RT can positively impact functional and metabolic changes in people with T2DM, this is the first meta-analysis that suggests that RT may benefit older adults (≥65 years) in the management of their disease. It is important to have accurate information for health care organizations to be able to integrate physical activity recommendations into their knowledge management strategies [5]. However, there are insufficient high quality studies (only 2 original RCTs, providing 3 records) that address the full impact of RT in older populations with T2DM. As such, confidence in conclusions based on the presented findings is limited. Furthermore, no study has included RT interventions with adults who were 80 years or older, despite this age group having the highest prevalence of T2DM [1]. Given the high prevalence and incidence of T2DM in geriatric populations [73], more research is needed to assess the potential benefits of RT for this age cohort. Also, some studies have suggested that there is an additive benefit from combining AT and RT exercises for adults with T2DM [9, 74, 75]. Future research should explore the effect of combined exercise training in populations who are at least 65 years old. 

Finally, the importance of conducting appropriate preexercise screens prior to implementing an RT or any exercise intervention cannot be overlooked [7]. This is of particular interest when working with older/clinical populations who may have various complications and comorbidities, resulting in absolute or relative contraindications to physical activity detailed in [7, page 276] and elaborated further by other researchers [18, 49]. 

 Measures that could provide additional insight into the benefits/risks of RT, such as muscle quality, fiber CSAs, changes in free fatty acid [17], and/or triglyceride concentrations [53], and medication reduction [13] were reported only in some papers and thus could not be meta-analyzed. As a result, a better understanding of the impact of RT in older adults with T2DM requires additional study. 

## 5. Limitations

 There are several limitations in this meta-analysis that are worth noting. Firstly, 68% of total participants from all three records are Hispanic. As such, the generalizability of the findings to different ethnic origins may be limited, due to the diversity of psychosocial and potentially genetic factors. 

Secondly, using the terms physical activity and exercise interchangeably may have varying outcome implications. For instance, studies that focus on physical activity may report different outcomes and result in alternate findings when compared to studies using a targeted training approach with predefined aims. 

Thirdly, the inclusion and exclusion criteria were developed to obtain the most relevant evidence for the population of older adults with type 2 diabetes, but with this strict criteria there is a risk that perhaps relevant studies that did not meet the specified inclusion requirements could have provided some additional insight for this paper. 

Fourthly, there is a risk of having a confounding variable effect by including Dunstan et al. [53], since their RT intervention was combined with a weight loss component. Thus, it is not possible to have a definitive conclusion about the independent effect the RT intervention might have had if it was not combined with the weight loss component. 

Despite these limitations, a rigorous approach has been undertaken to provide the first precise meta-analysis that assessed the currently available RCTs for RT effects on metabolic, musculoskeletal, and cardiovascular factors in adults 65 years or older with type 2 diabetes.

## 6. Conclusion

Although strong conclusions cannot be drawn from this meta-analysis, the potential role of RT to help older adults in the management of T2DM should be considered given the current trends in aging, obesity, and diabetes. In 2005, managing diabetes and its complications cost the Canadian acute healthcare system $5.6 billion [76], while in the US the current approximated annual cost is surpassing $134 billion dollars [1]. Also, these figures are excluding the personal costs endured by those with the disease and their families, associated with morbidity induced by various diabetic complications [1]. More recent statistics suggest that, factoring the cost of undiagnosed diabetes, prediabetes, and gestational diabetes, the total cost of diabetes in the US in 2007 totaled to $218 billion [77]. Considering that 26.9% of older adults in the US (approximately 10.9 million individuals) have diabetes [77], there ought to be specific and appropriately designed interventions for this cohort. Inclusion of RT in the management of T2DM has been recognized and supported by previous reviews [4, 12, 25, 47, 78, 79] and physical activity guidelines [27, 29, 34]. Future studies will help to confirm whether the metabolic benefits obtained with RT in younger populations could also positively impact older adults with T2DM, including the rapidly expanding population aged 80 years or more.

## Figures and Tables

**Table 1:** Outcome measures.

Body composition measures	Musculoskeletal measures	Type 2 diabetes process measures
Whole body lean tissue mass (kg) Whole body fat mass (kg)	Muscle strength (i) Upper body strength (ii) Lower body strength Muscle quality (defined as 1RM strength kg/unit lean body mass kg) Muscle fiber size (i) Type I cross sectional area (CSA) (*μ*m^2^) (ii) Type II CSA (*μ*m^2^)	Fasting glucose (mmol/L) Glycosylated hemoglobin (HbA1c) (%) Blood pressure Serum/fasting insulin (pmol/L) Lipids (i) Total cholesterol (mmol/L) (ii) HDL cholesterol (mmol) (iii) Triglycerides (iv) Free fatty acids (FFAs) (*μ*mol/L)

**Table 2:** Participant characteristics.

Source	Group (*n*)	Age (years)	Gender (M/F)	Whole body fat mass (kg)	BMI (kg/m^2^)	Diabetes duration (years)	HbA1c (%)	Fasting glucose (mmol/L)	Fasting insulin (pmol/L)
*Brooks et al. [17] Castaneda et al. [13]	Exercise 31 Control 31	66 ± 11.1 66 ± 5.6	10/21 19/12	35 ± 5.6 33.7 ± 13.4	30.9 ± 6.1 31.2 ± 5.6	8 ± 5.6 11 ± 5.6	8.7 ± 5.6 8.4 ± 1.7	8.79 ± 2.7 9.85 ± 3.8	116 ± 167.4 115 ± 176.9
Dunstan et al. [53]	Exercise 16 Control 13	67.6 ± 5.2 66.5 ± 5.3	10/6 6/7	33.1 ± 7.4 35.6 ± 6.8	31.5 ± 3.7 32.5 ± 3.8	7.6 ± 5.4 8.8 ± 7.9	8.1 ± 1 7.5 ± 1.1	9.5 ± 2.3 9.4 ± 2.1	132.9 ± 63 101.9 ± 25.8

All measures are provided as means ± SD.

*Brooks et al. [17] and Castaneda et al. [13] included the same cohort of participants.

**Table 3:** Study characteristics.

Study ID (reference number), PEDro score	Sample Size (*n*), intervention design	Intervention (duration, frequency, intensity, session duration, sets of reps, equipment: exercises)	Outcome measure (^¥^ *P* value)	Authors conclusion
*Brooks et al. [17] PEDro: 7	Exercise: *n* = 31 RT + SC Control: *n* = 31 SC	(i)16 weeks (ii) 3 d/week (iii) weeks 1–8: 60–80% of baseline 1RM; weeks 10–14: 70–80% of mid-study 1RM (iv) 45 min/session (5 min warmup; 5 min cooldown) (v) 3 sets of 8 reps (vi) 5 pneumatic machines: upper back, chest press, leg press, knee extension, and flexion	Whole-body lean tissue mass (0.04) Lower body muscle strength (<0.001) Upper body muscle strength (<0.001) Muscle quality (<0.001) Type I fiber CSA (0.04) Type II fiber CSA (0.04) HbA1c (<0.001) Fasting insulin (0.27) Fasting glucose (0.92) Whole body strength (0.0001)	16 weeks of RT resulted in musculoskeletal and metabolic improvements, and it is a mode of exercise worth considering as an adjunct to SC

*Castaneda et al. [13] PEDro: 6	Exercise: *n* = 31 RT + SC Control: *n* = 31 SC	(i) 16 weeks (ii) 3 d/week (iii) weeks 1–8: 60–80% of baseline 1RM; weeks 10–14: 70–80% of mid-study 1RM (iv) 45 min/session (5 min warmup; 5 min cooldown) (v) 3 sets of 8 reps (vi) 5 pneumatic machines: upper back, chest press, leg press, knee extension, and flexion	Whole body fat mass (0.26) Total cholesterol (0.59) LDL cholesterol (0.13) HDL cholesterol (0.46) Systolic BP (0.05) Diastolic BP (0.52)	RT was feasible among older adults with type 2 diabetes, and it resulted in improved metabolic control

Dunstan et al. [53] PEDro: 4	Exercise: *n* = 16 RT + WL Control: *n* = 13 WL	(i) 24 weeks (ii)3 d/week (iii) weeks 1-2: 50–60% 1RM; progress to: 75–85% 1RM (iv) 45 min/session (5 min warmup; 5 min cooldown) (v) 3 sets of 8–10 reps (minus abdominal curls) (vi) Free weights and multiple station weight machine; 9 exercises: bench press, leg extension, upright row, lateral pull down, standing leg curl with ankle weights, dumbbell seated shoulder press, dumbbell seated biceps curl, dumbbell biceps kickback, abdominal curls	Total cholesterol (N/A) LDL cholesterol (N/A) HDL cholesterol (N/A) HbA1c (<0.01) Fasting insulin (N/A) Fasting glucose (0.06) Systolic BP (<0.05) Diastolic BP (<0.05)	A 16-week progressive, high-intensity RT program was effective in improving glycemic control and muscle strength in older adults with T2DM

RT: resistance training; SC: standard care; d: days; min: minutes; sec: seconds; b/w: between; reps: repetitions; UE: upper extremity;

LE: lower extremity; CSA: cross sectional area; HbA1c: glycosylated hemoglobin; WL: weight loss).

*Brooks et al. [17] and Castaneda et al. [13] include the same intervention and participants but different outcome measures.

^¥^ *P* value reported by the authors.

**Table 4:** Heterogeneity for moderator variables.

Variable	*Q*-value	df (*Q*)	*P*-value
All disease process measures	42.387	19	0.002
BP	2.171	3	0.538
Fasting glucose	0.364	1	0.546
Fasting insulin	0.181	2	0.913
HbA1c	3.099	2	0.212
HDL	0.055	1	0.814
Total cholesterol and LDL	3.079	3	0.380
All musculoskeletal measures	31.313	11	0.001
Muscle quality	8.184	4	0.085
Muscle strength	2.675	2	0.262
Body composition	3.256	3	0.354

**Table 5:** PEDro rating details.

Study ID (PEDro score)	Random allocation	Concealed allocation	Baseline comparability	Blind subjects	Blind therapists	Blind assessors	Adequate followup	Intention-to-treat analysis	Between-group comparisons	Point estimates and variability
Brooks et al. (7) [17]	Yes	No	Yes	No	No	Yes	Yes	Yes	Yes	Yes
Castaneda et al. (6) [13]	Yes	No	No	No	No	Yes	Yes	Yes	Yes	Yes
Dunstan et al. (4) [53]	Yes	No	Yes	No	No	No	No	No	Yes	Yes

**Table 6:** Summary of resistance training effect on outcome measures.

Outcome		Hedge's *g*	*P* value	Effect description (statistical significance)	
Disease processes		−0.271^¥^	0.008	Medium (significant)	
BP (systolic/diastolic mmHg)		− 0.540	<0.001	Large (significant)	
HbA1c (%)		−0.463	0.145	Medium (not significant)	
Total and LDL cholesterol		−0.464	0.002	medium (significant)	
Fasting glucose		−0.121	0.559	Small (not significant)	
Fasting insulin		0.505	0.016	Medium (significant)	
HDL cholesterol		0.134	0.517	Small (not significant)	
Body composition		0.199	0.197	Small (not significant)	
Lean body mass		0.395	0.220	Small (not significant)	
Fat body mass		0.066	0.749	Small (not significant)	
Muscle strength		1.05	<0.001	Large (significant)	
Lower body muscle strength		1.415	<0.001	Large (significant)	
Upper body muscle strength		0.974	<0.001	Large (significant)	
Whole body muscle strength		0.802	0.002	Large (significant)	

*Further muscle measures		Exercise		Control	*P* value

Quality	Baseline	61 ± 27.8		51 ± 22.3	<0.001
	Final	100 ± 33.4		48 ± 22.3	
Type I CSA (*μ*m^2^)	Baseline	4068 ± 1425.3		4546 ± 1503.3	0.04
	Final	4928 ± 2071.2		4381 ± 1692.6	
Type II CSA (*μ*m^2^)	Baseline	3885 ± 1547.8		4330 ± 1926.4	0.04
	Final	4605 ± 1575.7		4201 ± 1870.8	

BP-blood pressure; HbA1c: glycosylated hemoglobin; LDL: low density lipoprotein cholesterol; HDL: high-density lipoprotein cholesterol; CSA: cross sectional area.

^¥^Negative values denote a decrease in the outcome measure (i.e., this is a positive effect, since a reduction in disease processes, such as lowered BP, LDL, and HBA1c, indicates an improvement in disease management).

*Further muscle measures were not entered into CMA; all values are means ± SE, taken from [17].
